# Association between triglyceride-glucose (TyG) index and diabetic foot ulcers in adult inpatients with type 2 diabetes at hospital admission

**DOI:** 10.3389/fcdhc.2026.1733439

**Published:** 2026-03-23

**Authors:** Alessandro De Stefano, Donatella Pastore, Marco Infante, Francesca Pacifici, Carmine Cardillo, Manfredi Tesauro, Francesca Schinzari, Federica Fazio, Massimiliano Caprio, Alessandro Terrinoni, Laura Di Renzo, Antonino De Lorenzo, Giulia Donadel, David Della-Morte

**Affiliations:** 1 Department of Systems Medicine, University of Rome Tor Vergata, Rome, Italy; 2 Department for the Promotion of Human Sciences and Quality of Life, San Raffaele Open University, Rome, Italy; 3 Section of Diabetes & Metabolic Disorders, Faculty of Medicine and Surgery, UniCamillus - Saint Camillus International University of Health Sciences, Rome/Venice, Italy; 4 Division of Cellular Transplantation, Diabetes Research Institute (DRI), Department of Surgery, University of Miami Miller School of Medicine, Miami, FL, United States; 5 Department of Translational Medicine and Surgery, Catholic University of the Sacred Heart, Rome, Italy; 6 Department of Aging, Fondazione Policlinico Universitario Agostino Gemelli IRCCS, Rome, Italy; 7 Geriatrics Unit, “Renato Dulbecco” University Hospital of Catanzaro, Catanzaro, Italy; 8 Laboratory of Cardiovascular Endocrinology, IRCCS San Raffaele, Rome, Italy; 9 Department of Biomedicine and Prevention, Section of Clinical Nutrition and Nutrigenomics, University of Rome Tor Vergata, Rome, Italy; 10 Department of Clinical Sciences and Translational Medicine, University of Rome Tor Vergata, Rome, Italy; 11 Department of Biomedical Sciences, Catholic University “Our Lady of Good Counsel”, Tirana, Albania; 12 Hepatology, Clinical Nutrition and Geriatrics Unit, Policlinico Tor Vergata (PTV) Hospital, University of Rome Tor Vergata, Rome, Italy; 13 Department of Neurology, Evelyn F. McKnight Brain Institute, University of Miami Miller School of Medicine, Miami, FL, United States

**Keywords:** DFU, diabetic foot ulcer, hospitalization, insulin resistance, lower-extremity amputations, TyG index, TyG-BMI index, type 2 diabetes

## Abstract

**Introduction:**

Diabetic foot ulcers (DFUs) are among the most serious complications of diabetes mellitus. Over the last years, the triglyceride-glucose (TyG) index has emerged as a surrogate marker of insulin resistance. We previously conducted a retrospective study on Albanian adult inpatients with type 2 diabetes (T2D), in which we showed that circulating triglycerides and fasting plasma glucose at hospital admission were among the most relevant independent variables associated with an increased risk of DFU in this population.

**Materials and methods:**

The present dual-center, retrospective case-control study assessed the relationship between the TyG index and the presence of DFU in a cohort of 497 adult inpatients (295 males; 202 females) with T2D, who were consecutively admitted to two Hospitals during different periods between 2014 and 2023. Patients with DFUs served as cases (DFU group; n=106), while patients without DFUs served as controls (non-DFU group; n=391).

**Results:**

Mean TyG index values were significantly higher in the DFU group than in the non-DFU group (5.02 ± 0.15 vs. 4.98 ± 0.09; p<0.001). Mean TyG-body mass index (BMI) index (TyG-BMI index) values were also significantly higher in the DFU group than in the non-DFU group (133.7 ± 23.5 vs. 126 ± 20.0; p=0.004). Based on the receiver operating characteristic (ROC) curve analysis, the TyG index showed a significant discriminative ability for the prediction of DFU (AUC = 0.61; 95% CI: 0.54-0.68; p<0.001), with a sensitivity of 38% and a specificity of 92% at the cut-off point of >5.08. In the multivariate logistic regression models, the TyG index was independently associated with the presence of DFU at hospital admission: odds ratio (OR) = 2.18; 95% confidence interval (CI): 1.68-2.83; p<0.0001 [model 2]; OR = 1.86; 95% CI: 1.27-2.71; p=0.001 [model 3].

**Conclusions:**

In conclusion, our study suggests that the TyG index may represent a valid prognostic biomarker among adult inpatients with T2D. Large prospective studies are needed to better clarify the predictive value of the TyG index for DFU, as well as its role as a marker of DFU severity among adult inpatients and outpatients with T2D.

## 1 Introduction

Type 2 diabetes (T2DM or T2D) is a chronic metabolic disease characterized by a gradual, non-autoimmune loss of adequate insulin secretion from pancreatic beta cells, often on the background of insulin resistance (1). T2D accounts for the majority (~90-95%) of all diagnosed cases of diabetes mellitus (1). According to the 11th Edition of the International Diabetes Federation (IDF) Diabetes Atlas (published in 2025), 589 million adults (aged 20–79 years) are affected by diabetes mellitus worldwide, and this number is projected to increase to 853 million by 2050 (2). Diabetic foot ulcers (DFUs) and their severe health consequences (particularly infection and amputation) are among the most serious complications of diabetes mellitus, being associated with a high risk of multiple hospitalizations, reduced quality of life, significant morbidity and mortality, along with high healthcare costs (3–7). It is estimated that approximately 18.6 million individuals worldwide are affected by a diabetic foot ulcer (DFU) each year (8). DFUs precede 80% of lower-extremity amputations (LEAs) in patients with diabetes mellitus (8). Diabetic foot infection is a common complication of DFUs, which can involve only the skin or superficial subcutaneous tissues, or it can extend to tendons, muscles, joints and bone (osteomyelitis) (9, 10). Notably, about 50-60% of DFUs become infected, with approximately 20% of moderate-to-severe DFU infections leading to LEAs (8). The 5-year mortality rate for patients with a DFU is about 30%, and it exceeds 70% for patients with a major amputation (8). Moreover, patients with DFUs exhibit a higher mortality rate compared to those with diabetes mellitus but without DFUs (231 deaths per 1000 person-years vs. 182 deaths per 1000 person-years, respectively) (8). DFUs result from a complex interaction of vascular, neurological and metabolic abnormalities, with diabetic peripheral neuropathy (DPN), foot deformities, peripheral arterial disease (PAD), ischemia, hyperglycemia, and/or infections contributing to tissue damage, delayed wound healing and major complications such as osteomyelitis and foot gangrene (11). Furthermore, DPN and PAD often coexist, thus leading to DFUs with a mixed neuropathic and ischemic etiology (neuroischemic foot ulcers) (12, 13). At the molecular level, chronic hyperglycemia favors the formation of advanced glycation end products (AGEs), increases oxidative stress, impairs macrophage shift from the pro-inflammatory M1 phenotype to the pro-healing M2 phenotype, hinders granulation tissue formation, and impairs angiogenesis, ultimately compromising normal wound healing processes (11, 14).

A key factor in the pathophysiology of T2D is insulin resistance, which is defined as a state of reduced insulin responsiveness of insulin-sensitive tissues, particularly liver, skeletal muscle and adipose tissue (15). Major factors contributing to the pathophysiology of insulin resistance include overweight/obesity, increased visceral adiposity and ectopic fat deposition, glucotoxicity, lipotoxicity due to excessive circulating concentrations of free fatty acids, physical inactivity, overnutrition, unhealthy eating habits, inflammation, gut dysbiosis, and genetic factors (16, 17). Insulin resistance is considered the major pathogenic driver of several chronic diseases, including metabolic dysfunction-associated steatotic liver disease (MASLD), atherosclerosis, T2D and metabolic syndrome (15, 18). Moreover, insulin resistance contributes to the development of microvascular and macrovascular complications of diabetes mellitus (19, 20), including diabetic foot syndrome (21). Therefore, early identification of insulin resistance can help adopt measures to prevent the onset of T2D or counteract the progression of the disease and the development of chronic complications (22–24). Over the last years, the triglyceride-glucose (TyG) index has emerged as a novel biomarker for the assessment of insulin resistance and cardiometabolic risk in patients at different stages across the cardiometabolic disease spectrum (25). Importantly, the TyG index has shown similar or even greater efficacy than conventional markers (such as the Homeostatic Model Assessment for Insulin Resistance [HOMA-IR]) in predicting cardiometabolic outcomes (25). A growing body of evidence has shown that the TyG index may also serve as a valid biomarker for the screening and follow-up of chronic complications of T2D, including cardiovascular disease, diabetic nephropathy, diabetic retinopathy, diabetic neuropathy and diabetic foot (24, 26).

Therefore, we conducted the present dual-center, retrospective case-control study to assess the relationship between the TyG index (as a surrogate marker of insulin resistance) and the presence of DFU in a cohort of adult inpatients with T2D at the time of admission to two Hospitals, namely: a) “Mother Teresa” University Hospital (Tirana, Albania); b) Policlinico Tor Vergata [PTV] Hospital, Units of Clinical Nutrition and Geriatrics, University of Rome Tor Vergata (Rome, Italy). A retrospective analysis conducted selectively on the Albanian cohort of the present study was already published elsewhere (27), in which we examined risk factors for DFUs (other than the TyG and TyG-BMI indexes) among adult inpatients with T2D and we showed that circulating triglycerides (TG) and fasting plasma glucose (FPG) at hospital admission were among the most relevant independent variables associated with an increased risk of DFU in a multivariate analysis performed by a logistic regression model [TG, odds ratio (OR)=7.48; 95% confidence interval (CI): 2.47-22.45; p=0.0004 - FPG, OR = 1.06; 95% CI: 1.03-1.08; p<0.0001] (27). In the present study, we expanded the previous study cohort by including Italian patients with T2D who were consecutively admitted to the Units of Clinical Nutrition and Geriatrics of Policlinico Tor Vergata (PTV) Hospital (University of Rome Tor Vergata; Rome, Italy) between January 2021 and December 2023. Moreover, we analyzed the TyG and TyG-BMI indexes, which were not assessed in the previously published study (27).

## 2 Materials and methods

### 2.1 Study design and participants

The present dual-center, retrospective case-control study was conducted on adult inpatients with T2D who were consecutively admitted to the “Mother Teresa” University Hospital (Tirana, Albania; period: June 2014 - January 2018) and to the Units of Clinical Nutrition and Geriatrics of Policlinico Tor Vergata (PTV) Hospital (University of Rome Tor Vergata; Rome, Italy; period: January 2021 - December 2023). The latest admission date for the Albanian cohort was December 2017. However, for one patient in the Albanian diabetic foot ulcer (DFU) cohort, data on glycated hemoglobin (HbA1c) measurement at the time of hospital admission (December 2017) were not available; therefore, for this patient, we considered the HbA1c measurement performed during the hospital stay (7 days after hospital admission; January 2018).

Inclusion criteria were the following: I) age ≥18 years; II) diagnosis of T2D based on patients’ past medical history and/or on the previous fulfillment of American Diabetes Association (ADA) diagnostic criteria [FPG ≥126 mg/dL; and/or 2-h plasma glucose during a 75-g oral glucose tolerance test ≥200 mg/dL; and/or HbA1c ≥6.5% (≥48 mmol/mol); and/or random plasma glucose ≥200 mg/dL accompanied by classic symptoms of hyperglycemia (such as polyuria, polydipsia, unexplained weight loss, polyphagia) or hyperglycemic crisis, together with the absence of islet autoantibodies; when unequivocal hyperglycemia is not present, the diagnosis of diabetes mellitus requires two abnormal results from distinct tests, which can be obtained at the same time, or from the same test obtained at two different time points] (1). Exclusion criteria were the following: I) pregnancy; II) lactation; III) active malignancy; IV) cognitive and neurodegenerative diseases; V) positive human immunodeficiency virus (HIV) and/or hepatitis B virus (HBV) and/or hepatitis C virus (HCV) serology testing results; VI) current use of immunosuppressive drugs and/or corticosteroids.

### 2.2 Data collection

We conducted a retrospective chart review to collect data regarding demographic, clinical and laboratory parameters of the study participants. Demographic, clinical and laboratory data were recorded in an anonymous database containing unambiguous and alphanumeric codes. By reviewing the patients’ medical records, we collected information regarding the following variables (referred to the time of hospital admission): age (years), gender, body mass index (BMI), cigarette smoking habit, duration of diabetes (years), past history of LEAs, and antidiabetic medication regimen. BMI was calculated by dividing the body weight in kilograms by the square of the body height in meters (kg/m^2^) (28). Based on data collected from medical records regarding the antidiabetic medication regimen, study participants were classified according to the use of insulin therapy alone, metformin therapy alone, metformin plus insulin combination therapy, and use of cardiorenal protective glucose-lowering drugs [glucagon-like peptide-1 receptor agonists (GLP-1 RA) and/or sodium-glucose cotransporter-2 inhibitors (SGLT2i)]. Based on patients’ past medical history and/or previous clinical and laboratory tests, we also collected information regarding the presence of the following additional comorbidities and chronic complications of diabetes (other than DFU) at the time of hospital admission: I) hypertension; II) diabetic retinopathy; III) diabetic peripheral neuropathy; IV) diabetic nephropathy. With respect to our previously published study (27), hypertension was defined as a systolic blood pressure (SBP) value ≥130 mmHg and/or as a diastolic blood pressure (DBP) value ≥80 mmHg, in accordance with the recent AHA/ACC/AANP/AAPA/ABC/ACCP/ACPM/AGS/AMA/ASPC/NMA/PCNA/SGIM guideline for the prevention, detection, evaluation and management of high blood pressure in adults (29). According to the international guidelines (29), blood pressure values were based on an average of two or more blood pressure measurements obtained on two or more separate occasions (starting from the time of hospital admission).

Study participants were divided into two groups based on the presence or absence of DFUs, as follows: I) patients with DFU, who served as cases (DFU group); II) patients without DFU, who served as controls (non-DFU group). DFU was defined as a full-thickness skin lesion requiring more than 14 days for healing (30). At the time of hospital admission, DFUs were also classified according to the Meggitt‐Wagner DFU classification system, which estimates wound severity based on wound depth and tissue viability (31, 32). According to the Meggitt‐Wagner DFU classification system, DFUs were classified as follows: grade 1 (ulcer of the superficial skin layers/subcutaneous tissue that does not involve deeper tissues); grade 2 (ulcer that extends into ligament, tendon, joint capsule, or deep fascia); grade 3 (ulcer that extends into deeper tissues such as bone and/or joint, with abscess formation or osteomyelitis); grade 4 (forefoot gangrene); grade 5 (gangrene that involves more than two thirds of the foot) (31, 32).

The presence of DFU infection was evaluated based on clinical and laboratory features indicative of local inflammation/infection and/or purulence, such as wound exudate, wound odor, swelling, surrounding cellulitis, “crackling” sensation on palpation, positive probe-to-bone test, presence of tissue necrosis, leukocytosis, and/or fever (4, 9, 10, 33). A minor LEA was considered as a lower limb resection through or distal to the ankle, while a major LEA was considered as a lower limb resection proximal to the ankle (34).

We evaluated laboratory parameters obtained from blood samples collected in the morning after an overnight fast of at least 8 hours (on the day following hospital admission), namely: FPG, HbA1c, serum creatinine, TG, total cholesterol (TC), and high-density lipoprotein cholesterol (HDL-C). Low-density lipoprotein cholesterol (LDL-C) was calculated using the Friedewald equation (35). The TyG index was calculated using the following equation: ln [fasting triglycerides (mg/dL) × fasting plasma glucose (mg/dL)]/2; as it has previously been described (36). The TyG-BMI index was calculated - as it has previously been described (37) - using the following formula: TyG index × BMI. The estimated glomerular filtration rate (eGFR; expressed in mL/min/1.73 m^2^) was calculated using the 2021 Chronic Kidney Disease Epidemiology Collaboration (CKD-EPI) equation (38).

### 2.3 Statistical analysis

Descriptive statistics are presented as mean (± standard deviation [SD]) for continuous variables with a Gaussian distribution, and as frequency (counts and percentages; n, %) for categorical variables. The Kolmogorov-Smirnov test was used to assess data distribution. Comparison between the DFU group and the non-DFU group was performed using an unpaired t-test or the Mann-Whitney U test for continuous variables, as appropriate, and the Fisher’s exact test for categorical variables and percentages. The correlation between the TyG index and the other study variables was assessed using the Spearman’s correlation analysis or the Pearson’s correlation analysis, as appropriate. A non-parametric Spearman’s rank correlation matrix was used to evaluate pairwise correlations between the variables assessed in the study.

A univariate linear regression analysis was performed to evaluate the relationship between the TyG index and the other variables assessed in the study. Two multiple linear backward regression analyses (conducted in the entire study cohort and in the DFU group, respectively) were performed to evaluate the relationship between the TyG index and the other variables assessed in the study. The independent variables analyzed in the aforementioned multiple linear regression analyses were the following: age, gender (female), BMI (kg/m^2^), cigarette smoking, FPG, HbA1c, serum creatinine, eGFR, TC, LDL-C, HDL-C, TG, triglyceride-to-high-density lipoprotein cholesterol (TG/HDL-C) ratio, TyG index, TyG-BMI index, duration of diabetes, hypertension, SBP, DBP, diabetic retinopathy, diabetic peripheral neuropathy, diabetic nephropathy, use of insulin therapy alone, use of metformin therapy alone, use of metformin plus insulin combination therapy, use of GLP-1 RA and/or SGLT2i.

Univariate and multivariate analyses of independent predictors of DFU were performed using a backward logistic regression model. The risk of DFU development was estimated using the OR with the 95% confidence interval (CI). The univariate logistic regression model (model 1) included the following variables: age, gender (female), BMI (kg/m^2^), cigarette smoking, FPG, HbA1c, serum creatinine, eGFR, TC, LDL-C, HDL-C, TG, TG/HDL-C ratio, TyG index, TyG-BMI index, duration of diabetes, hypertension, SBP, DBP, diabetic retinopathy, diabetic peripheral neuropathy, diabetic nephropathy, use of insulin therapy alone, use of metformin therapy alone, use of metformin plus insulin combination therapy, use of GLP-1 RA and/or SGLT2i. The multivariate logistic regression model (model 2) included the following independent variables: cigarette smoking, HbA1c, serum creatinine, eGFR, TG/HDL-C ratio, TyG index, TyG-BMI index, duration of diabetes, hypertension, SBP, use of insulin therapy alone, use of metformin therapy alone, use of metformin plus insulin combination therapy, and use of GLP-1 RA and/or SGLT2i. Receiver operating characteristic (ROC) curve analysis of the multivariate logistic regression model (model 2) was employed to calculate the AUC and to assess the model’s discriminative ability for the prediction of DFU. In another multivariate logistic regression model (model 3), age and gender were also included (in addition to the other variables that were already included in the model 2). After the backward elimination process, variables that did not reach statistical significance were excluded from the multivariate logistic regression models. FPG, TG and BMI were not included in the multivariate logistic regression models, since these models already included the TyG index and the TyG-BMI index as composite markers calculated from FPG, TG and BMI.

In the DFU group, mean TyG index values were compared between DFU subgroups established based on the ulcer grade (defined according to the Meggitt‐Wagner DFU classification system) and on the past history of LEAs. The one-way analysis of variance (one-way ANOVA) was performed for the comparisons between the DFU subgroups based on the ulcer grade, while the Mann-Whitney U test was performed for the comparisons between the DFU subgroups based on the past history of LEAs.

In all statistical analyses, a p-value <0.05 was considered statistically significant. Statistical analyses were performed using GraphPad Prism 10 (GraphPad Software Inc., San Diego, CA, USA) and MedCalc version 23.2.1 (MedCalc Software Ltd; Ostend, Belgium).

### 2.4 Informed consent and ethical approval

At the time of hospital admission, all study participants and/or their legal guardians provided written informed consent to anonymous data collection, analysis and publication for research purposes. The study was conducted according to the principles of the Declaration of Helsinki. The study was approved by the Ethics Committee of the Catholic University “Our Lady of Good Counsel” (Tirana, Albania; registration number: M-FP3:125/17; 2017) and by the Ethics Committee of the University of Rome Tor Vergata (Rome, Italy; registration number: 115.25 CET2 PTV; 2025).

## 3 Results

This study included a total of 497 adult inpatients with T2D (295 males; 202 females). Of these 497 patients, 482 patients were admitted to the “Mother Teresa” University Hospital (Tirana, Albania), while 15 patients were admitted to the Units of Clinical Nutrition and Geriatrics of Policlinico Tor Vergata (PTV) Hospital (University of Rome Tor Vergata, Rome, Italy). Mean age of the study participants was 55 ± 11 years (age range: 29–93 years). Of the total 497 patients, 106 (65 males; 41 females) were affected by DFU (DFU group), while 391 (230 males; 161 females) were not affected by DFU (non-DFU group). The main causes of hospital admission among study participants included occurrence of DFU and DFU infection in the DFU group, and elevated blood glucose values (random plasma glucose ≥200 mg/dL on the day of hospital admission) in the non-DFU group. Participants’ demographic, clinical and laboratory parameters are shown in **Table 1**.

**Table 1:** Demographic, clinical and laboratory parameters of the study participants.

Variable	Entire study cohort n=497	DFU group (cases) n=106	Non-DFU group (controls) n=391	P-value
Age (years)*	55 ± 11 [29-93]	54 ± 13 [30-93]	55 ± 10 [29-87]	0.16
Gender (M/F) (n, %)	295 (59.3%)/202 (40.7%)	65 (61.3%)/41 (38.7%)	230 (58.8%)/161 (41.2%)	0.66
BMI (kg/m^2^)	25.6 ± 4.3	26.6 ± 4.92	25.3 ± 4.14	**0.02**
Cigarette smokers (n, %)	303 (61%)	93 (87.8%)	210 (53.7%)	**<0.001**
FPG (mg/dL)	197 ± 39.9	245.5 ± 55.7	183.8 ± 19.42	**<0.001**
HbA1c (%)	8.2 ± 1.6	9.6 ± 2.34	7.8 ± 1	**<0.001**
Serum creatinine (mg/dL)	1.2 ± 0.5	1.35 ± 0.4	1.19 ± 0.5	**<0.001**
eGFR (mL/min/1.73 m^2^)	67.4 ± 30.4	59.5 ± 23.6	69.6 ± 31.7	**0.004**
TC (mg/dL)	176.4 ± 25.9	169.6 ± 25.2	178.2 ± 25.8	**0.001**
LDL-C (mg/dL)	109.1 ± 19.7	108.3 ± 18.5	109.3 ± 20.1	0.28
HDL-C (mg/dL)	44.4 ± 3.9	41.5 ± 4	45.2 ± 3.4	**<0.001**
TG (mg/dL)	114.5 ± 20.6	99 ± 17.2	118.8 ± 19.4	**<0.001**
TG/HDL-C ratio	2.56 ± 0.35	2.37 ± 0.25	2.62 ± 0.36	**<0.001**
TyG index	4.99 ± 0.11	5.02 ± 0.15	4.98 ± 0.09	**<0.001**
TyG-BMI index	127.7 ± 21	133.7 ± 23.5	126 ± 20.0	**0.004**
Duration of diabetes (years)	9.7 ± 7.2	10.4 ± 7.7	9.5 ± 7	0.39
Hypertension (n, %)	184 (37%)	56 (52.8%)	128 (32.8%)	**<0.001**
SBP (mmHg)	131.4 ± 16	135.9 ± 15.8	130 ± 15.8	**<0.001**
DBP (mmHg)	76.3 ± 11.6	78 ± 10.8	76 ± 11.8	0.49
Diabetic retinopathy (n, %)	166 (33.4%)	64 (60.3%)	102 (26.1%)	**<0.001**
Diabetic peripheral neuropathy (n, %)	174 (35%)	78 (73.6%)	96 (24.6%)	**<0.001**
Diabetic nephropathy (n, %)	165 (33.3%)	47 (44.3%)	118 (30.2%)	**0.007**
Use of insulin therapy alone (n, %)**	258 (52%)	30 (28%)	228 (58.3%)	**<0.001**
Use of metformin therapy alone (n, %)	125 (25.1%)	58 (55%)	67 (17.1%)	**<0.001**
Use of metformin plus insulin combination therapy (n, %)**	99 (19.9%)	16 (15%)	83 (21.3%)	0.17
Use of GLP-1 RA and/or SGLT2i (n, %)	15 (3%)	2 (2%)	13 (3.3%)	0.75

Data are presented as mean (± standard deviation [SD]) for continuous variables, and as frequency (counts and percentages; n, %) for categorical variables. Comparison between the DFU group (cases) and the non-DFU group (controls) was performed using an unpaired t-test or the Mann-Whitney U test for continuous variables, as appropriate, and the Fisher’s exact test for categorical variables. A p-value <0.05 was considered statistically significant. Statistically significant p-values are shown in bold. The eGFR (expressed in mL/min/1.73 m^2^) was calculated using the 2021 Chronic Kidney Disease Epidemiology Collaboration (CKD-EPI) equation. *The age range is shown in square brackets. **In the entire study cohort, all patients on insulin therapy alone were using a basal-bolus insulin regimen (multiple daily subcutaneous insulin injections), while patients on metformin plus insulin combination therapy were using a long-acting insulin analog (basal insulin in combination with metformin). BMI, body mass index; DBP, diastolic blood pressure; DFU, diabetic foot ulcer; eGFR, estimated glomerular filtration rate; F, females; FPG, fasting plasma glucose; GLP-1 RA, glucagon-like peptide-1 receptor agonists; HbA1c, glycated hemoglobin; HDL-C, high-density lipoprotein cholesterol; LDL-C, low-density lipoprotein cholesterol; M, males; SBP, systolic blood pressure; SGLT2i, sodium-glucose co-transporter-2 inhibitors; TC, total cholesterol; TG, triglycerides; TyG index, triglyceride-glucose index; TyG-BMI index, triglyceride glucose-BMI index.

According to the Meggitt‐Wagner DFU classification system, the DFU group (at the time of hospital admission; n=106) included 19 (17.9%) patients with a grade 1 ulcer, 67 patients (63.2%) with a grade 2 ulcer, and 20 patients (18.9%) with a grade 3 ulcer. At the time of hospital admission, none of the patients in the DFU group exhibited foot gangrene (grade 4 or grade 5 DFUs according to the Meggitt‐Wagner DFU classification system).

At hospital admission, all subjects in the DFU group (n=106) showed clinical signs of ulcer infection: patients with a grade 1 ulcer (n=19; 17.9%) showed clinical signs of infection limited to the superficial layers of the skin, patients with a grade 2 ulcer (n=67; 63.2%) showed clinical signs of infection involving the subcutaneous tissue, and patients with a grade 3 ulcer (n=20; 18.9%) showed clinical signs of infection involving the bone. None of the patients in the DFU group (n=106) had a past history of major LEAs, while 41 patients (38.7%) had a past history of minor LEAs and 65 patients (61.3%) did not have a past history of LEAs.

**Figure 1:**
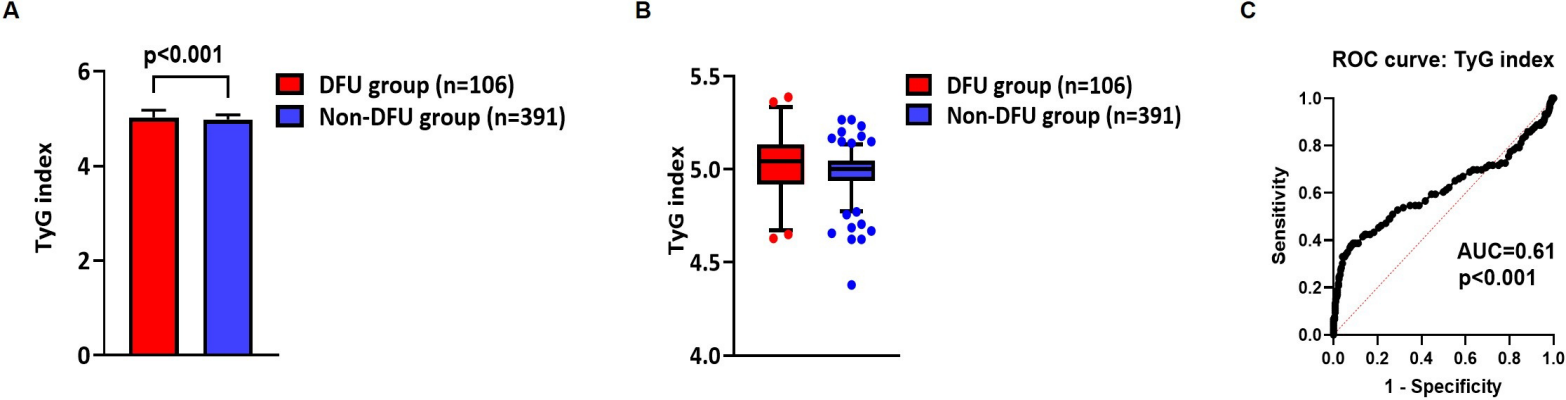
Comparison of mean **(A)** and median **(B)** TyG index values between the DFU group and the non-DFU group; ROC curve analysis showing the discriminative ability of the TyG index for the prediction of DFU **(C)**. In panel A, error bars represent the standard deviation (SD). In panel B, boxes represent the 25th and 75th percentiles, lines inside the boxes represent median values, whiskers represent the upper and lower adjacent values, and dots represent outliers outside the whiskers. AUC, area under the curve; DFU, diabetic foot ulcer; TyG index, triglyceride-glucose index; ROC curve, receiver operating characteristic curve.

Mean TyG index values were slightly - but significantly - higher in the DFU group than in the non-DFU group (5.02 ± 0.15 vs. 4.98 ± 0.09; p<0.001) (**Table 1**; **Figure 1A**). Median TyG index values were 5.04 (25th percentile: 4.92; 75th percentile: 5.13) in the DFU group and 5.00 (25th percentile: 4.93; 75th percentile: 5.04) in the non-DFU group (**Figure 1B**). Moreover, mean TyG-BMI index values were significantly higher in the DFU group than in the non-DFU group (133.7 ± 23.5 vs. 126 ± 20.0; p=0.004) (**Table 1**). ROC curve analysis showed that the TyG index had a significant discriminative ability for the prediction of DFU (area under the curve [AUC]=0.61; 95% CI: 0.54-0.68; p<0.001), with a sensitivity of 38% and a specificity of 92% at the cut-off point of >5.08 (**Figure 1C**). ROC curve analysis showed similar results even when the TyG was calculated with the equation in which the product of fasting triglycerides and fasting plasma glucose was divided by 2 inside the logarithm, as it has also been reported in the literature (39): AUC = 0.60; 95% CI: 0.55-0.64; p=0.008 (with a sensitivity of 36% and a specificity of 91% at the cut-off point of >9.49). As compared to participants in the non-DFU group, subjects in the DFU group also exhibited significantly higher mean values of BMI, FPG, HbA1c, serum creatinine and SBP, as well as significantly lower mean values of eGFR, TC, HDL-C, TG, and TG/HDL-C ratio (**Table 1**). Additionally, there was a significantly higher proportion of cigarette smokers and individuals affected by hypertension, diabetic retinopathy, diabetic peripheral neuropathy and diabetic nephropathy in the DFU group compared with the non-DFU group (**Table 1**). In the entire study cohort, all patients on insulin therapy alone were using a basal-bolus insulin regimen (multiple daily subcutaneous insulin injections), while patients on metformin plus insulin combination therapy were using a long-acting insulin analog (basal insulin in combination with metformin). There was a significantly lower proportion of patients on insulin therapy alone and a significantly higher proportion of patients on metformin therapy alone in the DFU group compared with the non-DFU group (**Table 1**). At hospital admission, the daily metformin dose ranged from 500 mg to 2500 mg.

There was no significant difference between the DFU group and the non-DFU group in terms of age, proportion of males and females, mean LDL-C value, mean DBP value, duration of diabetes, use of metformin plus insulin combination therapy, and use of cardiorenal protective glucose-lowering drugs (GLP-1 RA and/or SGLT2i) (**Table 1**). GLP-1 RA included once-daily subcutaneous liraglutide, once-weekly subcutaneous dulaglutide and once-daily oral semaglutide, whereas SGLT2i included dapagliflozin, empagliflozin and canagliflozin.

### 3.1 TyG index in DFU and non-DFU groups: correlation analysis, linear regression analysis and multiple linear regression analysis

**Figure 2:**
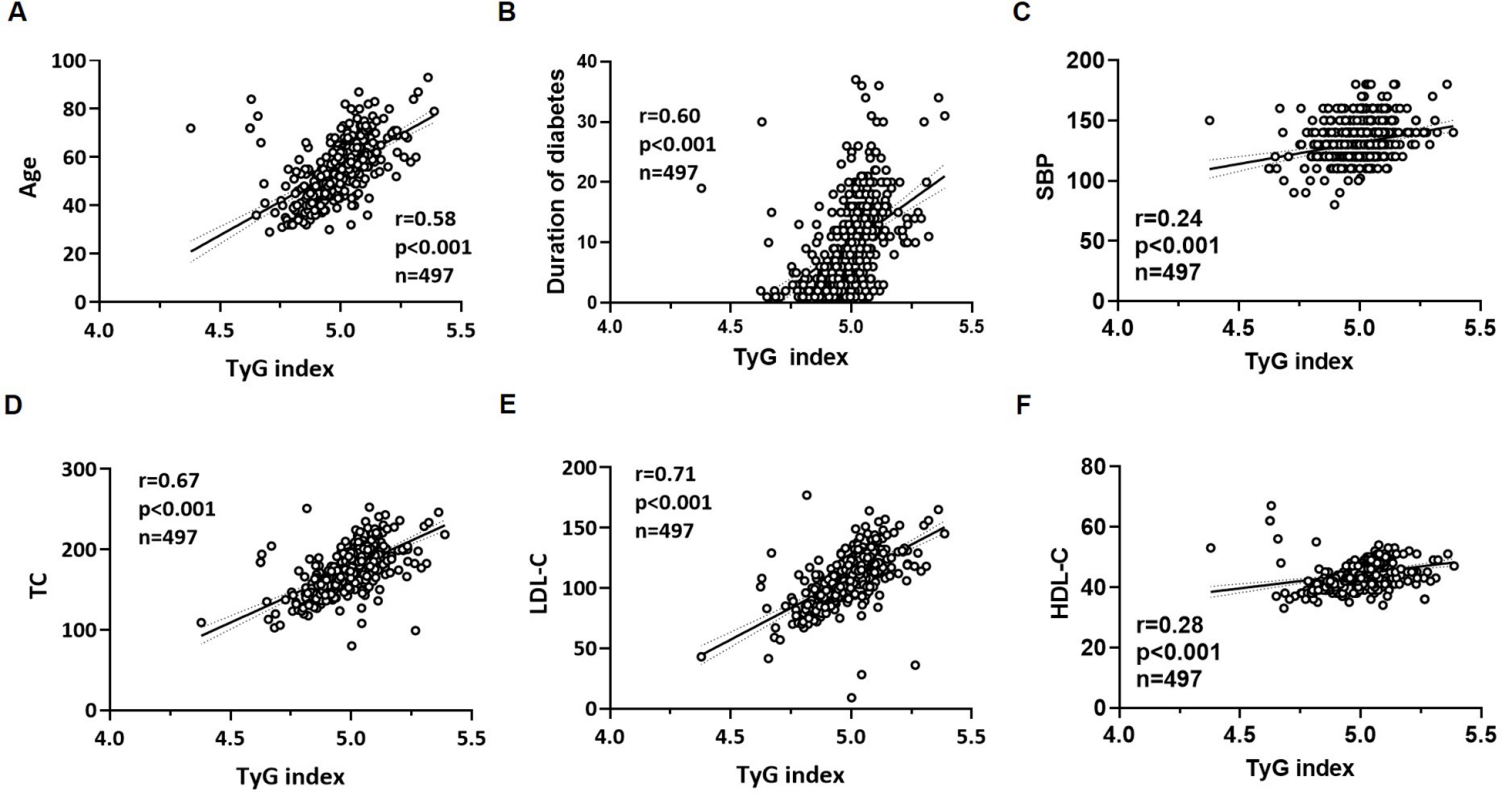
Scatter plots regarding the correlation analysis between the TyG index and age **(A)**, duration of diabetes **(B)**, SBP **(C)**, TC **(D)**, LDL-C **(E)** and HDL-C **(F)**. The p-value (p) indicates the statistical significance in the univariate linear regression analysis. HDL-C, high-density lipoprotein cholesterol; LDL-C, low-density lipoprotein cholesterol; r, correlation coefficient; SBP, systolic blood pressure; TC, total cholesterol; TyG index, triglyceride-glucose index.

**Figure 3:**
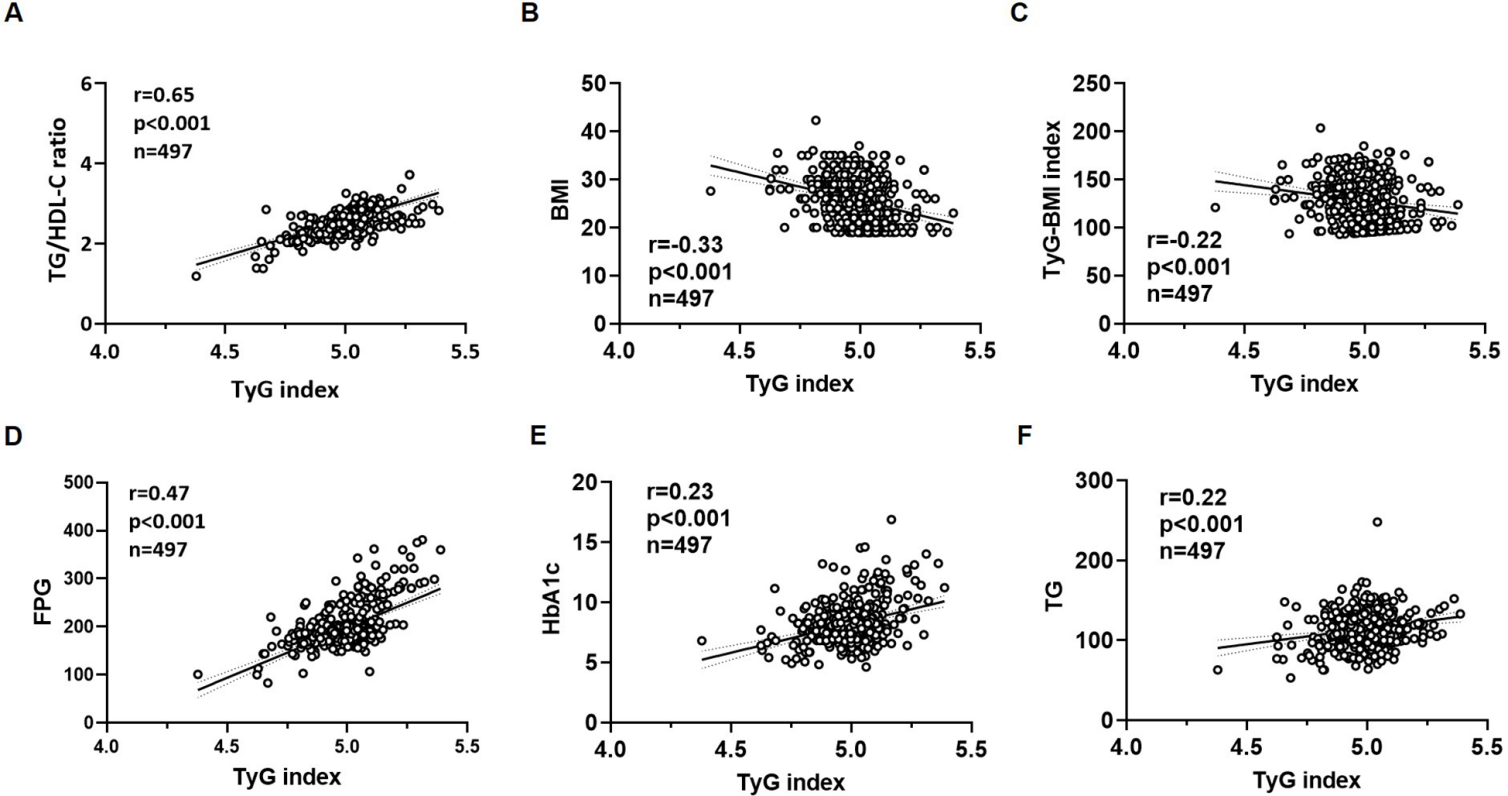
Scatter plots regarding the correlation analysis between the TyG index and TG/HDL-C ratio **(A)**, BMI **(B)**, TyG-BMI index **(C)**, FPG **(D)**, HbA1c **(E)** and TG **(F)**. The p-value (p) indicates the statistical significance in the univariate linear regression analysis. BMI, body mass index; FPG, fasting plasma glucose; HbA1c, glycated hemoglobin; r, correlation coefficient; TG, triglycerides; TG/HDL-C ratio, triglyceride-to-high-density lipoprotein cholesterol ratio; TyG index, triglyceride-glucose index; TyG-BMI index, triglyceride glucose-BMI index.

A nonparametric Spearman’s rank correlation matrix was used to evaluate pairwisecorrelations between the variables assessed in the study (results are shown in **Supplementary Figure 1**). The univariate linear regression analysis showed that the TyG index had a significant positive linear correlation with age (r=0.58; p<0.001), duration of diabetes (r=0.60; p<0.001), SBP (r=0.24; p<0.001), TC (r=0.67; p<0.001), LDL-C (r=0.71; p<0.001), HDL-C (r=0.28; p<0.001) (**Figure 2**). The univariate linear regression analysis also showed that the TyG index had a significant positive linear correlation with FPG (r=0.47; p<0.001), TG/HDL-C ratio (r=0.65; p<0.001), HbA1c (r=0.23; p<0.001), TG (r=0.22; p<0.001) (**Figure 3**), hypertension (r=0.17; p<0.001), cigarette smoking (r=0.14; p<0.001), and diabetic retinopathy (r=0.21; p<0.001). On the other hand, the univariate linear regression analysis showed that the TyG index had a significant negative linear correlation with BMI (r=-0.33; p<0.001), TyG-BMI index (r=-0.22; p<0.001) (**Figure 3**), use of insulin therapy alone (r=-0.12; p=0.005), use of metformin therapy alone (r=-0.02; p=0.005), and use of metformin plus insulin combination therapy (r=-0.19; p<0.001). There was no significant correlation between the TyG index and the remaining variables (female gender, DBP, serum creatinine, eGFR, diabetic peripheral neuropathy, use of GLP-1 RA and/or SGLT2i, and diabetic nephropathy).

**Table 2** and **Table 3** report the statistically significant results of the multiple linear regression analyses investigating the association between the TyG index and all the other variables assessed in the entire study cohort (**Table 2**) and in the DFU group (**Table 3**).

**Table 2:** Statistically significant results from the multiple linear regression analysis investigating the association between the TyG index and the other variables assessed in the entire study cohort (including the DFU group and the non-DFU group).

Independent variables	β	95% CI	P-value	r_partial_
BMI	-0.1668	-0.1716 to -0.1621	**<0.001**	-0.95
Age	0.00105	0.00065 to 0.00144	**<0.001**	0.23
FPG	0.00017	0.00011 to 0.00023	**<0.001**	0.25
HbA1c	0.00185	0.00066 to 0.00303	**0.002**	0.14
TC	-0.00952	-0.01353 to -0.0055	**<0.001**	-0.20
LDL-C	0.00951	0.00564 to 0.01339	**<0.001**	0.21
HDL-C	0.00975	0.00557 to 0.01393	**<0.001**	0.20
TG	0.00200	0.00117 to 0.00283	**<0.001**	0.21
TyG-BMI index	0.03345	0.03250 to 0.03440	**<0.001**	0.95
Use of GLP-1 RA and/or SGLT2i	-0.02354	-0.04115 to -0.0059	**0.009**	-0.12

The table shows only the statistically significant results obtained from the multiple linear regression analysis.

A p-value <0.05 was considered statistically significant. The symbol β indicates the beta coefficient (regression coefficient), while r_partial_ indicates the partial correlation coefficient. Statistically significant p-values are shown in bold. 95% CI, 95% confidence interval; BMI, body mass index; DFU, diabetic foot ulcer; FPG, fasting plasma glucose; GLP-1 RA, glucagon-like peptide-1 receptor agonists; HbA1c, glycated hemoglobin; HDL-C, high-density lipoprotein cholesterol; LDL-C, low-density lipoprotein cholesterol; SGLT2i, sodium-glucose cotransporter-2 inhibitors; TC, total cholesterol; TG, triglycerides; TyG index, triglyceride-glucose index; TyG-BMI index, triglyceride glucose-BMI index.

**Table 3:** Statistically significant results from the multiple linear regression analysis investigating the association between the TyG index and the other variables assessed in the DFU group.

Independent variables	β	95% CI	P-value	r_partial_
BMI	-0.06359	-0.07613 to -0.05106	**<0.001**	-0.72
FPG	0.001354	0.001212 to 0.00149	**<0.001**	0.89
TC	0.001278	0.0009869 to 0.00156	**<0.001**	0.66
TG/HDL-C ratio	0.1207	0.08459 to 0.1568	**<0.001**	0.56
TyG-BMI index	0.01268	0.01019 to 0.01518	**<0.001**	0.72
Use of insulin therapy alone*	0.007958	0.001504 to 0.01441	**0.02**	0.24
Use of metformin plus insulin combination therapy*	-0.01118	-0.01908 to -0.00328	**0.006**	-0.28

The table shows only the statistically significant results obtained from the multiple linear regression analysis.

A p-value <0.05 was considered statistically significant. The symbol β indicates the beta coefficient (regression coefficient), while r_partial_ indicates the partial correlation coefficient. Statistically significant p-values are shown in bold. *In the entire study cohort, all patients on insulin therapy alone were using a basal-bolus insulin regimen (multiple daily subcutaneous insulin injections), while patients on metformin plus insulin combination therapy were using a long-acting insulin analog (basal insulin in combination with metformin). 95% CI, 95% confidence interval; BMI, body mass index; DFU, diabetic foot ulcer; FPG, fasting plasma glucose; TC, total cholesterol; TG/HDL-C ratio, triglyceride-to-high-density lipoprotein cholesterol ratio; TyG index, triglyceride-glucose index; TyG-BMI index, triglyceride glucose-BMI index.

In the multiple linear backward regression analysis performed on the entire study cohort, the participants’ age was significantly and positively associated with the TyG index (β=0.00105; p<0.001) (**Table 2**). Moreover, LDL-C and HDL-C were both significantly and positively correlated with the TyG index (β=0.00951, p<0.001 - β=0.00975, p<0.001; respectively). Conversely, TC (β=-0.00952; p<0.001) and BMI (β=-0.1668; p<0.001) were significantly and negatively correlated with the TyG index. As expected, TyG-BMI index (β=0.03345; p<0.001), TG (β=0.00200; p<0.001) and FPG (β=0.00017; p<0.001) were significantly and positively correlated with the TyG index. HbA1c was also significantly and positively correlated with the TyG index (β=0.00185; p=0.002). The use of GLP-1 RA and/or SGLT2i was significantly and inversely correlated with the TyG index (β=-0.02354; p=0.009) (**Table 2**).

In the multiple linear backward regression analysis conducted only in the DFU group (**Table 3**), variables that were significantly and positively correlated with the TyG index included FPG (β=0.001354; p<0.001), TC (β=0.001278; p<0.001), TG/HDL-C ratio (β=0.127; p<0.001), TyG-BMI index (β=0.01268; p<0.001), and use of insulin therapy alone (β=0.007958; p=0.02). On the other hand, BMI and the use of metformin plus insulin combination therapy were significantly and negatively correlated with the TyG index (β=-0.06359; p<0.001 - β =-0.01118; p=0.006) (**Table 3**).

### 3.2 Univariate and multivariate logistic regression analyses for the assessment of the relationship between the TyG index and the presence of DFU at hospital admission

Univariate and multivariate logistic regression analyses were performed to explore the relationship between the TyG index values and the presence of DFU at the time of hospital admission. **Table 4** shows the results of the logistic regression analyses examining the association between the TyG index and the presence of DFU at hospital admission. In the univariate analysis (model 1), the TyG index was significantly associated with increased odds of presenting with DFU at hospital admission [OR = 1.25 (95% CI: 1.15-1.37); p<0.001].

**Table 4:** Univariate and multivariate logistic regression analyses examining the association between the TyG index and the presence of DFU.

Independent variable	Univariate analysis (model 1)		Multivariate analysis (model 2)	
	OR (95% CI)	p-value	OR (95% CI)	p-value
Age (years)	0.99 (0.97-1.00)	0.29	–	
Gender (female)	0.90 (0.58-1.40)	0.64	–	
BMI (kg/m^2^)	1.07 (1.02-1.13)	**0.005**	–	
Cigarette smoking	5.67 (3.12-10.28)	**<0.001**	6.51 (2.45-17.24)	**<0.001**
FPG (mg/dL)	1.05 (1.04-1.06)	**<0.001**	–	
HbA1c (%)	2.40 (1.86-2.70)	**<0.001**	1.61 (1.09-2.39)	**0.01**
Serum creatinine (mg/dL)	2.13 (1.33-3.40)	**0.001**	0.10 (0.01-0.75)	**0.02**
eGFR (mL/min/1.73 m^2^)	0.98 (0.97-0.99)	**0.002**	0.95 (0.92-0.98)	**0.007**
TC (mg/dL)	0.98 (0.97-0.99)	**0.003**	–	
LDL-C (mg/dL)	0.99 (0.98-1.00)	0.73	–	
HDL-C (mg/dL)	0.72 (0.67-0.78)	**<0.001**	–	
TG (mg/dL)	0.94 (0.93-0.95)	**<0.001**	–	
TG/HDL-C ratio	0.06 (0.02-0.13)	**<0.001**	0.00 (0.00-0.00)	**<0.0001**
TyG index	1.25 (1.15-1.37)	**<0.001**	2.18 (1.68-2.83)	**<0.0001**
TyG-BMI index	1.01 (1.00-1.02)	**<0.001**	–	
Duration of diabetes (years)	1.01 (0.98-1.05)	0.22	1.22 (1.12-1.33)	**<0.0001**
Hypertension	2.27 (1.47-3.50)	**<0.001**	–	
SBP (mmHg)	1.02 (1.00-1.03)	**0.001**	1.02 (1.00-1.05)	**0.04**
DBP (mmHg)	1.00 (0.98-1.02)	0.39	–	
Diabetic retinopathy	4.30 (3.70-6.80)	**<0.001**	–	
Diabetic peripheral neuropathy	8.50 (5.20-14.00)	**<0.001**	–	
Diabetic nephropathy	1.84 (1.18-2.86)	**<0.001**	–	
Use of insulin therapy alone*	0.20 (0.13-0.32)	**<0.001**	–	
Use of metformin therapy alone	3.45 (2.15-5.50)	**<0.001**	–	
Use of metformin plus insulin combination therapy*	0.70 (0.30-1.23)	0.22	5.00 (1.55-16.06)	**0.006**
Use of GLP-1 RA and/or SGLT2i	0.56 (0.12-2.15)	0.49	0.00 (0.000-0.001)	**<0.0001**

The univariate logistic regression model (model 1) included the following variables: age, gender (female), BMI (kg/m^2^), cigarette smoking, FPG, HbA1c, serum creatinine, eGFR, TC, LDL-C, HDL-C, TG, TG/HDL-C ratio, TyG index, TyG-BMI index, duration of diabetes, hypertension, SBP, DBP, diabetic retinopathy, diabetic peripheral neuropathy, diabetic nephropathy, use of insulin therapy alone, use of metformin therapy alone, use of metformin plus insulin combination therapy, use of GLP-1 RA and/or SGLT2i. The multivariate logistic regression model (model 2) included the following independent variables: cigarette smoking, HbA1c, serum creatinine, eGFR, TG/HDL-C ratio, TyG index, TyG-BMI index, duration of diabetes, hypertension, SBP, use of insulin therapy alone, use of metformin therapy alone, use of metformin plus insulin combination therapy, and use of GLP-1 RA and/or SGLT2i. After the backward elimination process, TyG-BMI index, hypertension, use of insulin therapy alone, and use of metformin therapy alone were excluded from the multivariate logistic regression model due to the lack of statistical significance. A p-value <0.05 was considered statistically significant. Statistically significant p-values are shown in bold. The eGFR (expressed in mL/min/1.73 m^2^) was calculated using the 2021 Chronic Kidney Disease Epidemiology Collaboration (CKD-EPI) equation. *In the entire study cohort, all patients on insulin therapy alone were using a basal-bolus insulin regimen (multiple daily subcutaneous insulin injections), while patients on metformin plus insulin combination therapy were using a long-acting insulin analog (basal insulin in combination with metformin). Abbreviations: 95% CI, 95% confidence interval; BMI, body mass index; DBP, diastolic blood pressure; eGFR, estimated glomerular filtration rate; FPG, fasting plasma glucose; GLP-1 RA, glucagon-like peptide-1 receptor agonists; HbA1c, glycated hemoglobin; HDL-C, high-density lipoprotein cholesterol; LDL-C, low-density lipoprotein cholesterol; OR, odds ratio; SBP, systolic blood pressure; SGLT2i, sodium-glucose cotransporter-2 inhibitors; TC, total cholesterol; TG, triglycerides; TyG index, triglyceride-glucose index; TyG-BMI index, triglyceride glucose-BMI index.

In the multivariate logistic regression analysis (model 2), TyG-BMI index, hypertension, use of insulin therapy alone and use of metformin therapy alone were excluded from the model due to the lack of statistical significance. In the multivariate logistic regression analysis (model 2), the TyG index was significantly and independently associated with increased odds of presenting with DFU at hospital admission [OR = 2.18 (95% CI: 1.68-2.83); p<0.0001] (**Table 4**). Other independent variables that were included in the multivariate logistic regression model (model 2) and remained significantly and independently associated with increased odds of presenting with DFU at hospital admission were the following: cigarette smoking [OR = 6.51 (95% CI: 2.45-17.24); p<0.001], HbA1c [OR = 1.61 (95% CI: 1.09-2.39); p=0.01], duration of diabetes [OR = 1.22 (95% CI: 1.12-1.33); p<0.0001], SBP [OR = 1.02 (95% CI: 1.00-1.05); p=0.04], and use of metformin plus insulin combination therapy [OR = 5.00 (95% CI: 1.55-16.06); p=0.006] (**Table 4**). Moreover, variables that were significantly and independently associated with reduced odds of presenting with DFU at hospital admission were the following: serum creatinine [OR = 0.10 (95% CI: 0.01-0.75); p=0.02], eGFR [OR = 0.95 (95% CI: 0.92-0.98); p=0.007], TG/HDL-C ratio [OR = 0.00 (95% CI: 0.00-0.00); p<0.0001], and use of GLP-1 RA and/or SGLT2i [OR = 0.00 (95% CI: 0.000-0.001); p<0.0001] (**Table 4**). Importantly, the combination of the independent variables included in the multivariatelogistic regression model (model 2) showed a high discriminative ability (AUC = 0.96; 95% CI:0.94-0.98; p<0.001) to predict the presence of DFU (**Supplementary Figure 2**). Remarkably, the TyG index remained significantly and independently associated with increased odds of presenting with DFU at hospital admission [OR = 1.86 (95% CI: 1.27-2.71); p=0.001] even in a multivariate logistic regression model (model 3) that also included age and gender (in addition to the other variables that were already included in the model 2). The overall significance levels of multivariate logistic regression models 2 and 3 were defined by the following p-values: p<0.0001 and p=0.0001, respectively.

### 3.3 Subgroup analysis on the association between the TyG index, ulcer grade (Meggitt‐Wagner classification system) and past history of minor LEAs

**Figure 4:**
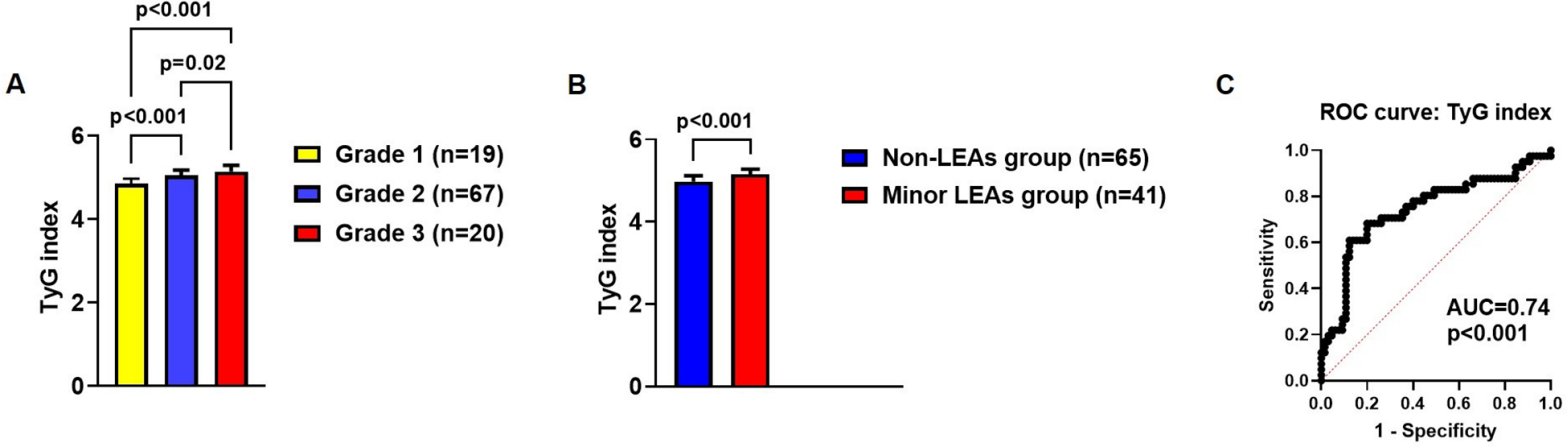
Mean TyG index values in different subgroups of patients with DFU, who were classified based on ulcer grade (as defined by the Meggitt‐Wagner DFU classification system; panel **A**) and past history of minor LEAs **(B)**; ROC curve analysis assessing the discriminative ability of the TyG index for the prediction of minor LEAs in the DFU group **(C)**. In panels A and B, error bars represent the standard deviation (SD). AUC, area under the curve; DFU, diabetic foot ulcer; LEAs, lower-extremity amputations; ROC curve, receiver operating characteristic curve; TyG index, triglyceride-glucose index.

**Figure 4** shows the TyG index values in different subgroups of patients with DFU, who were classified based on ulcer grade (Meggitt‐Wagner DFU classification system) and past history of minor LEAs. Mean TyG index values were significantly higher in patients with a grade 3 ulcer (5.13 ± 0.15) compared to patients with grade 1 (4.85 ± 0.12) and grade 2 (5.04 ± 0.12) ulcers: grade 3 group vs. grade 1 group, p<0.001; grade 3 group vs. grade 2 group, p=0.02 (**Figure 4A**). Mean TyG index values were also significantly higher in patients with a grade 2 ulcer (5.04 ± 0.12) compared to patients with a grade 1 ulcer (4.85 ± 0.12) [p<0.001] (**Figure 4A**). Furthermore, mean TyG index values were significantly higher (p<0.001) in patients with a past history of minor LEAs (5.15 ± 0.12) compared to those without a past history of LEAs (4.97 ± 0.14) (**Figure 4B**). The ROC curve analysis revealed that the TyG index had a significant discriminative ability for the prediction of minor LEAs in the DFU group [AUC = 0.74 (95% CI: 0.64-0.85); p<0.001], with a sensitivity of 84% and a specificity of 80% at the cut-off point of >5.07 (**Figure 4C**). ROC curve analysis showed similar results even when the TyG was calculated with the equation in which the product of fasting triglycerides and fasting plasma glucose was divided by 2 inside the logarithm, as it has also been reported in the literature (39): AUC = 0.77; 95% CI: 0.68-0.84; p<0.001 (with a sensitivity of 71% and a specificity of 81% at the cut-off point of >9.46).

## 4 Discussion

This study found that adult inpatients with T2D and DFU, as compared to adult inpatients with T2D but without DFU, exhibited significantly higher mean values of TyG index and TyG-BMI index at the time of hospital admission. According to the ROC curve analysis, the TyG index showed a significant discriminative ability for the prediction of DFU [AUC = 0.61 (95% CI: 0.54-0.68); p<0.001], with a sensitivity of 38% and a specificity of 92% at the cut-off point of >5.08. Patients with DFU also showed significantly higher mean values of BMI, FPG, HbA1c, serum creatinine and SBP, as well as significantly lower mean values of eGFR, TC, HDL-C, TG and TG/HDL-C ratio compared to adult inpatients with T2D but without DFU. Additionally, there was a significantly higher proportion of cigarette smokers and a significantly higher prevalence of hypertension and microvascular complications of diabetes (diabetic retinopathy, diabetic peripheral neuropathy and diabetic nephropathy) in patients with DFU compared to those without DFU. These findings are in line with previous literature, as it is known that DFU is often associated with poor glucose control, hypertension, overweight/obesity, dyslipidemia, hypertension, cigarette smoking, poor medication adherence, and other macrovascular and microvascular complications of diabetes (40–49).

In the DFU group, as compared to the non-DFU group, there were also a significantly lower proportion of patients on insulin therapy alone (basal-bolus insulin therapy) and a significantly higher proportion of patients on metformin therapy alone. This finding may be explained by the fact that patients with DFU had a worse glucose control than patients without DFU partly as a consequence of therapeutic inertia and non-optimized glucose-lowering therapy (metformin monotherapy).

The multiple linear regression analysis conducted on the entire study cohort documented that the TyG index was significantly and positively correlated with age, LDL-C, HDL-C, HbA1c, TyG-BMI index, TG (as expected) and FPG (as expected), while it was significantly and negatively correlated with TC, BMI and with the use of GLP-1 RA and/or SGLT2i. The multiple linear regression analysis conducted only in the DFU group documented that the TyG index was significantly and positively correlated with FPG (as expected), TC, TG/HDL-C ratio, TyG-BMI index, and with the use of insulin therapy alone, while it was significantly and negatively correlated with BMI and with the use of metformin plus insulin (long-acting insulin) combination therapy. These findings suggest that TyG index may serve a surrogate marker of glucose and lipid control in adult inpatients with T2D and DFUs.

A possible explanation for the inverse relationship between the TyG index and BMI observed in our study cohort is represented by the subcutaneous fat depots’ function as reservoirs that store excess energy in the form of triglycerides and prevent or mitigate the detrimental effects of hypertriglyceridemia and excessive circulating concentrations of free fatty acids (50). In addition, subjects with higher BMI values may have a better nutritional status (51).

The fact that the TyG index was significantly and positively associated with the TG/HDL-C ratio in the DFU group is in line with the established role of the TG/HDL-C ratio as an additional surrogate marker of insulin resistance (52) and as an indicator of atherogenic dyslipidemia (53). As expected, the TyG index was also significantly and positively associated with the TyG-BMI index in the entire study cohort and selectively in the DFU group, further corroborating the role of the TyG-BMI index as an additional surrogate marker of insulin resistance (54).

According to our multivariate logistic regression analysis (model 2), the TyG index was significantly and independently associated with increased odds of presenting with DFU at hospital admission [OR = 2.18 (95% CI: 1.68-2.83); p<0.0001] in a cohort of 497 hospitalized T2D patients (106 patients with DFU; 391 patients without DFU). Moreover, the TyG index remained significantly and independently associated with increased odds of presenting with DFU at hospital admission [OR = 1.86 (95% CI: 1.27-2.71); p=0.001] even in a multivariate logistic regression model (model 3) that also included age and gender (in addition to the other variables that were already included in the model 2).

In the multivariate logistic regression analysis (model 2), other variables that remained significantly and independently associated with increased odds of presenting with DFU at hospital admission were the following: cigarette smoking [OR = 6.51 (95% CI: 2.45-17.24); p<0.001], HbA1c [OR = 1.61 (95% CI: 1.09-2.39); p=0.01], duration of diabetes [OR = 1.22 (95% CI: 1.12-1.33); p<0.0001], SBP [OR = 1.02 (95% CI: 1.00-1.05); p=0.04], use of metformin plus insulin (long-acting insulin) combination therapy [OR = 5.00 (95% CI: 1.55-16.06); p=0.006]. These findings are in line with those reported by other studies in the scientific literature (43, 45, 46, 55, 56). On the other hand, variables that were significantly and independently associated with reduced odds of presenting with DFU at hospital admission in model 2 were the following: serum creatinine [OR = 0.10 (95% CI: 0.01-0.75); p=0.02], eGFR [OR = 0.95 (95% CI: 0.92-0.98); p=0.007], TG/HDL-C ratio [OR = 0.00 (95% CI: 0.00-0.00); p<0.0001], and use of GLP-1 RA and/or SGLT2i [OR = 0.00 (95% CI: 0.000-0.001); p<0.0001]. However, the lack of information on medications other than glucose-lowering drugs (including lipid-lowering drugs and antihypertensive drugs) prevents the clinical interpretation of the latter findings (particularly those regarding the TG/HDL-C ratio). With regard to the use of cardiorenal protective glucose-lowering drugs, the retrospective study design (with data available only at a single time point) and the low number of patients using the GLP-1 RA and/or SGLT2i (n=15; 2 patients with DFU and 13 patients without DFU; all patients from the Italian study cohort) precludes drawing reliable conclusions regarding the relationship between the use of such drugs and protection against the development of DFU in our study cohort. Moreover, there was no significant difference between the DFU group and the non-DFU group in terms of use of GLP-1 RA and/or SGLT2i. Similarly, it is not possible to draw conclusions regarding the significant association observed between the use of metformin plus insulin (long-acting insulin) combination therapy and the increased odds of presenting with DFU at hospital admission.

Finally, we found that mean TyG index values were significantly higher in patients with Meggitt‐Wagner grade 3 DFUs (deeper DFUs) compared to those with Meggitt‐Wagner grade 1 and grade 2 DFUs (5.13 ± 0.15 vs. 4.85 ± 0.12 and 5.04 ± 0.12, respectively). Furthermore, mean TyG index values were significantly higher in patients with a past history of minor LEAs (5.15 ± 0.12) compared to those observed in patients without a past history of LEAs (4.97 ± 0.14). Remarkably, the ROC curve analysis revealed that the TyG index showed a significant discriminative ability for the prediction of minor LEAs in the DFU group [AUC = 0.74 (95% CI: 0.64-0.85); p<0.001], with a sensitivity of 84% and a specificity of 80% at the cut-off point of >5.07. Thus, the latter findings seem to indicate that the TyG index may also serve as a marker of disease severity and disease burden in patients with DFU, as it has also been observed by Chen and colleagues (57).

Other studies have previously investigated the relationship between the TyG index and DFUs, yielding diverging results (57–59). Chen et al. (57) conducted a retrospective, single-center study on 1059 patients with T2D to investigate if the TyG index is related to the severity of DFUs. After adjusting for potential confounders (including age, gender, BMI, hemoglobin, albumin, smoking, alcohol use, peripheral artery disease, HbA1c, serum lipid levels, duration of diabetes ≥ 10 years, eGFR), the highest TyG index tertile (>7.90), as compared to the lowest TyG index tertile (≤7.28), was significantly associated with the presence of severe DFUs [OR = 1.506 (95% CI: 1.079-2.103); p=0.016] (57). A Spanish case-control study conducted on 70 adults (33 cases with diabetes; 37 controls without diabetes) demonstrated that the TyG index, together with the phase angle, was strongly linked to the risk of diabetic foot (58). A Peruvian retrospective study conducted on 162 adults with diabetic foot showed that a TyG index value greater than 9.4 was associated with an increased risk of amputation after one year (60). Moreover, it has been shown that elevated TyG index values are independently associated with an increased risk of sudden cardiac death among inpatients with T2D and DFU (61).

Conversely, Li et al. (59) conducted a cross-sectional study on 8866 hospitalized adults with diabetes mellitus, finding that the TyG index was significantly lower in patients with DFUs compared to those without DFUs. Moreover, the logistic regression analysis conducted in this study revealed that the TyG index quartiles 2 (Q2; 8.66-9.16), 3 (Q3; 9.16-9.69) and 4 (Q4; 9.69-12.94), as compared to the TyG index quartile 1 (Q1; 6.18-8.66), were significantly (p<0.001) and inversely associated with the risk of diabetic foot: Q2 group (OR = 0.75; 95% CI: 0.60-0.93), Q3 group (OR = 0.58; 95% CI: 0.45-0.75) and Q4 group (OR = 0.40; 95% CI: 0.28-0.55) (59). The ROC curve analysis showed a discriminative ability of the TyG index for the presence of DFU with an AUC of 0.661 (95% CI: 0.642-0.680; p<0.001) (59). To explain the counterintuitive relationship between the TyG index and DFUs observed in this study, Li et al. (59) speculated that the TyG index may not only serve as a surrogate marker of insulin resistance, but it may also represent a marker of hypoglycemia, which has been associated with an increased risk of amputations in patients with diabetic foot (62).

### 4.1 Study limitations

We acknowledge that the present study has various limitations that prevent its generalizability and restrict the clinical interpretation of the observed findings. These limitations include the retrospective study design and the lack of relevant information, such as the exact time of onset of DFUs and microvascular complications of diabetes, prevalence of dyslipidemia, use and type of lipid-lowering drugs, use and type of antihypertensive drugs, wound swab culture test results, ankle-brachial index values, and prevalence of macrovascular complications of diabetes. Another limitation of the present study was the lack of information on inflammatory markers in study participants at the time of hospital admission, since TyG index values in the DFU group may have been influenced by the presence of DFU infection. Therefore, a causal relationship between the TyG index values and the development of DFU cannot be established based on our study findings. However, it is worth highlighting that the advantages of TyG index as a surrogate marker of insulin resistance and related comorbidities in patients with diabetes mellitus include its low cost and ease of measurement, as well as the fact that it is not dependent on fasting insulinemia, which in turn can be affected by exogenous insulin therapy (unlike other markers of insulin resistance such as HOMA-IR and quantitative insulin sensitivity check index [QUICKI]) (63).

## 5 Conclusions

In conclusion, this study suggests a possible role of insulin resistance (as measured by the TyG index) in the pathophysiology of DFU and DFU-related complications. In particular, our findings suggest that the TyG index may serve as a useful biomarker for identifying T2D patients at high risk of developing DFUs and DFU-related complications, potentially allowing for targeted prevention and management strategies. Of note, the TyG index and its related indexes (such as the TyG-BMI index) may represent valid prognostic biomarkers among adult inpatients with T2D and established DFUs. Therefore, large prospective studies are certainly needed to better clarify the predictive value of the TyG index for DFU, as well as its role as a marker of DFU severity among adult inpatients and outpatients with T2D. Additionally, it would be useful to conduct such studies in diverse populations, since the TyG index cut-off values for the prediction of DFU and DFU severity may vary based on gender and ethnic differences. Finally, randomized controlled trials are also warranted to establish whether therapeutic interventions aimed to reduce the TyG index values are effective in the prevention and treatment of DFUs among adult inpatients and outpatients with T2D.

## Data Availability

The original contributions presented in the study are included in the article/**Supplementary Material**. Further inquiries can be directed to the corresponding author.
