# Supplementary material for: Association between triglyceride-glucose (TyG) index and diabetic foot ulcers in adult inpatients with type 2 diabetes at hospital admission
Source: Front Clin Diabetes Healthc. 2026 Mar 23;7:1733439. doi: 10.3389/fcdhc.2026.1733439 (PMC13050659; doi:10.3389/fcdhc.2026.1733439)
Supplement: Supplementary file 1 [file DataSheet1.docx]

Supplementary Material

# Supplementary Figures


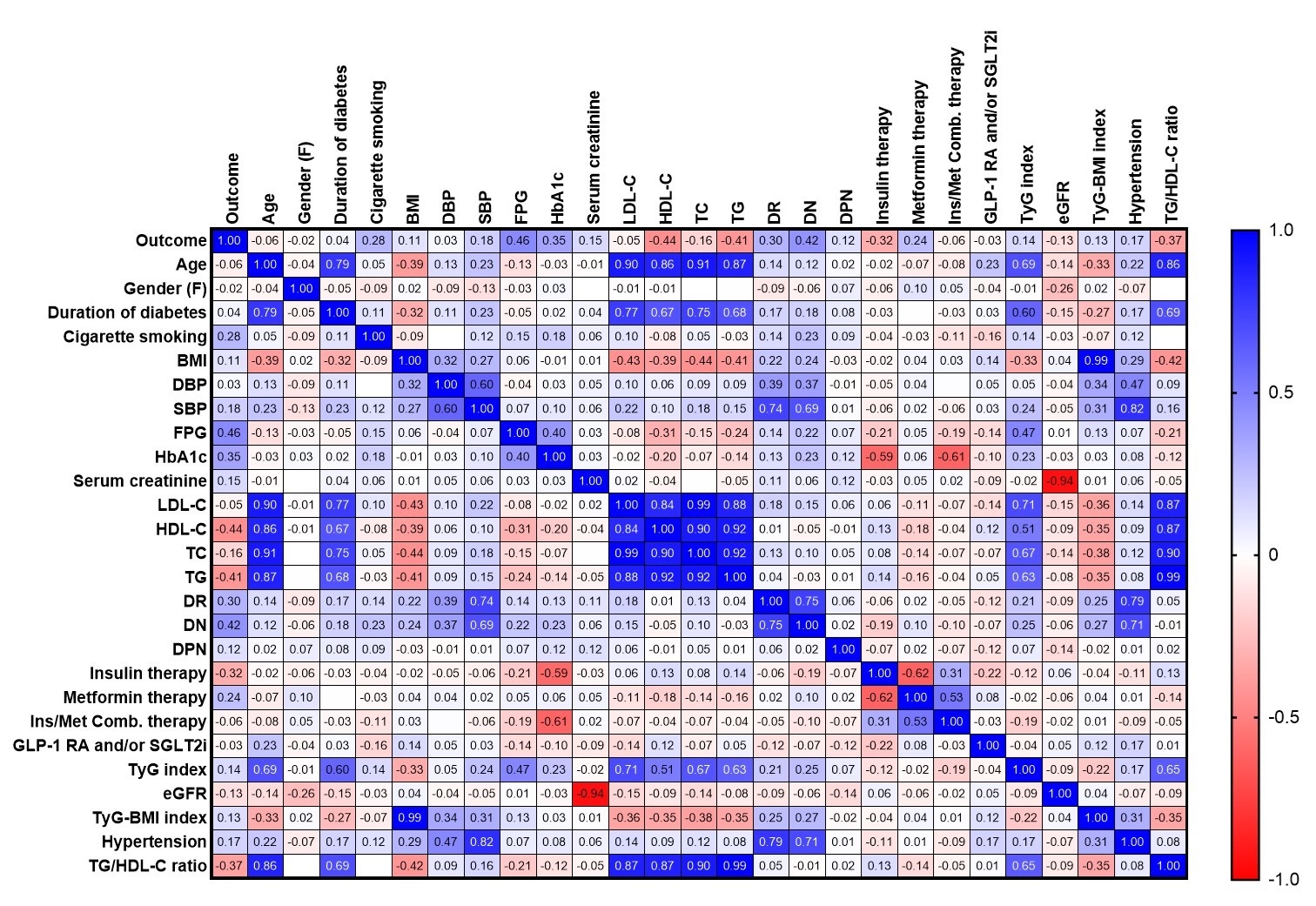


**Supplementary Figure S1. Heat-map Spearman’s rank-order correlation analysis performed in the entire study population to evaluate pairwise correlations between the variables assessed in the study.** A p-value <0.05 was considered statistically significant. The eGFR (expressed in mL/min/1.73 m^2^) was calculated using the 2021 Chronic Kidney Disease Epidemiology Collaboration (CKD-EPI) equation. Abbreviations: BMI, body mass index; DBP, diastolic blood pressure; DN, diabetic nephropathy; DPN, diabetic peripheral neuropathy; DR, diabetic retinopathy; eGFR, estimated glomerular filtration rate; FPG, fasting plasma glucose; Gender (F), gender (female); GLP-1 RA, glucagon-like peptide-1 receptor agonists; HbA1c, glycated hemoglobin; HDL-C, high-density lipoprotein cholesterol; Ins/Met Comb. Therapy, use of metformin plus insulin (long-acting insulin) combination therapy; Insulin therapy, use of insulin therapy alone; LDL-C, low-density lipoprotein cholesterol; Metformin therapy, use of metformin therapy alone; SBP, systolic blood pressure; SGLT2i, sodium-glucose cotransporter-2 inhibitors; TC, total cholesterol; TG, triglycerides; TG/HDL-C ratio, triglyceride-to-high-density lipoprotein cholesterol ratio; TyG index, triglyceride-glucose index; TyG-BMI index, triglyceride glucose-BMI index.


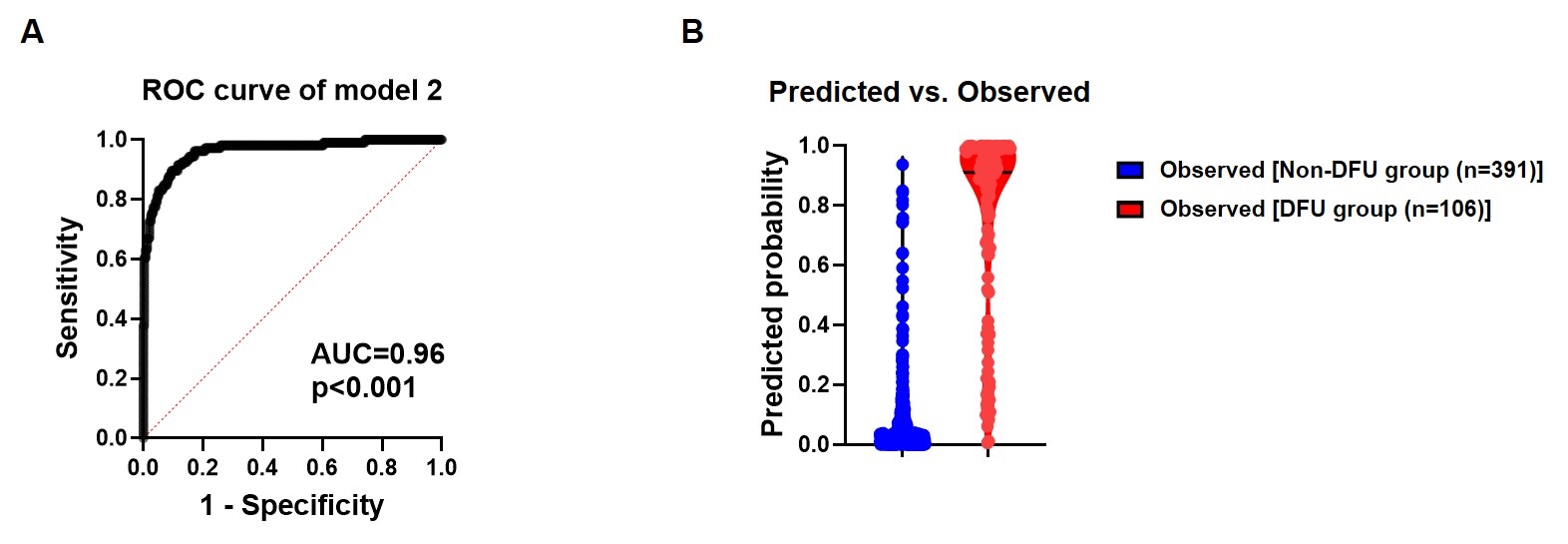


**Supplementary Figure S2. Receiver operating characteristic (ROC) curve and predicted probability distributions of the multivariate logistic regression model (model 2).** **Panel (A)**: Receiver operating characteristic (ROC) curve analysis of the multivariate logistic regression model (model 2) employed to calculate the area under the curve (AUC) and to assess the model’s discriminative ability for the prediction of DFU. **Panel (B)**: Violin plot showing the distribution of predicted probabilities for participants in the non-DFU group and in the DFU group.
